# Supplementary material for: Modeling glioblastoma heterogeneity as a dynamic network of cell states
Source: Mol Syst Biol. 2021 Sep 16;17(9):e10105. doi: 10.15252/msb.202010105 (PMC8444284; doi:10.15252/msb.202010105)
Supplement: Supplementary file 6 — Source Data for Figure 5 [file MSB-17-e10105-s004.zip › Figure5A_sourcedata/GSEA_3017/hallmarks_stateC.GseaPreranked.1621934520804/HALLMARK_G2M_CHECKPOINT.html]

Details for gene set HALLMARK\_G2M\_CHECKPOINT[GSEA]

|  || Dataset | state13017 |
| Phenotype | NoPhenotypeAvailable |
| Upregulated in class | na\_pos |
| GeneSet | HALLMARK\_G2M\_CHECKPOINT |
| Enrichment Score (ES) | 0.58296436 |
| Normalized Enrichment Score (NES) | 3.2398973 |
| Nominal p-value | 0.0 |
| FDR q-value | 0.0 |
| FWER p-Value | 0.0 |
Table: GSEA Results Summary

  

Fig 1: Enrichment plot: HALLMARK\_G2M\_CHECKPOINT      
 Profile of the Running ES Score & Positions of GeneSet Members on the Rank Ordered List

  

| PROBE | GENE SYMBOL | GENE\_TITLE | RANK IN GENE LIST | RANK METRIC SCORE | RUNNING ES | CORE ENRICHMENT || 1 | EZH2 |  |  | 14 | 0.574 | 0.0327 | Yes |
| 2 | CHAF1A |  |  | 27 | 0.494 | 0.0609 | Yes |
| 3 | ORC6 |  |  | 39 | 0.450 | 0.0864 | Yes |
| 4 | TMPO |  |  | 49 | 0.432 | 0.1138 | Yes |
| 5 | TOP2A |  |  | 52 | 0.426 | 0.1528 | Yes |
| 6 | SMC2 |  |  | 63 | 0.386 | 0.1737 | Yes |
| 7 | E2F1 |  |  | 67 | 0.380 | 0.2064 | Yes |
| 8 | NASP |  |  | 75 | 0.371 | 0.2311 | Yes |
| 9 | SMC4 |  |  | 78 | 0.365 | 0.2641 | Yes |
| 10 | GINS2 |  |  | 83 | 0.360 | 0.2930 | Yes |
| 11 | STMN1 |  |  | 85 | 0.357 | 0.3269 | Yes |
| 12 | BARD1 |  |  | 86 | 0.357 | 0.3625 | Yes |
| 13 | FOXN3 |  |  | 94 | 0.347 | 0.3849 | Yes |
| 14 | NUSAP1 |  |  | 100 | 0.340 | 0.4100 | Yes |
| 15 | MCM3 |  |  | 114 | 0.324 | 0.4195 | Yes |
| 16 | SMC1A |  |  | 125 | 0.312 | 0.4331 | Yes |
| 17 | HMGN2 |  |  | 126 | 0.312 | 0.4643 | Yes |
| 18 | FBXO5 |  |  | 130 | 0.308 | 0.4897 | Yes |
| 19 | KIF15 |  |  | 136 | 0.302 | 0.5110 | Yes |
| 20 | LMNB1 |  |  | 142 | 0.293 | 0.5315 | Yes |
| 21 | SLC12A2 |  |  | 148 | 0.288 | 0.5515 | Yes |
| 22 | MYBL2 |  |  | 164 | 0.278 | 0.5529 | Yes |
| 23 | MAD2L1 |  |  | 174 | 0.270 | 0.5640 | Yes |
| 24 | CDK1 |  |  | 191 | 0.255 | 0.5614 | Yes |
| 25 | E2F3 |  |  | 194 | 0.252 | 0.5830 | Yes |
| 26 | SQLE |  |  | 293 | -0.274 | 0.4381 | No |
| 27 | MYC |  |  | 421 | -0.336 | 0.2484 | No |
| 28 | MT2A |  |  | 566 | -0.575 | 0.0527 | No |
Table: GSEA details [plain text format]

  

Fig 2: HALLMARK\_G2M\_CHECKPOINT: Random ES distribution      
 Gene set null distribution of ES for **HALLMARK\_G2M\_CHECKPOINT**
